# Transcriptomic Profiling of Skeletal Muscle Reveals Candidate Genes Influencing Muscle Growth and Associated Lipid Composition in Portuguese Local Pig Breeds

**DOI:** 10.3390/ani11051423

**Published:** 2021-05-16

**Authors:** André Albuquerque, Cristina Óvilo, Yolanda Núñez, Rita Benítez, Adrián López-Garcia, Fabián García, Maria do Rosário Félix, Marta Laranjo, Rui Charneca, José Manuel Martins

**Affiliations:** 1 MED-Mediterranean Institute for Agriculture, Environment and Development, Instituto de Investigação e Formação Avançada & Universidade de Évora, Pólo da Mitra, Ap. 94, 7006-554 Évora, Portugal; mlaranjo@uevora.pt; 2 Departamento de Mejora Genética Animal, Instituto Nacional de Investigación y Tecnología Agraria y Alimentaria (INIA), 28040 Madrid, Spain; ovilo@inia.es (C.Ó.); nunez.yolanda@inia.es (Y.N.); rmbenitez@inia.es (R.B.); adrian.lopez@inia.es (A.L.-G.); fabian.garcia@inia.es (F.G.); 3 MED & Departamento de Fitotecnia, Escola de Ciências e Tecnologia, Universidade de Évora, Pólo da Mitra, Ap. 94, 7006-554 Évora, Portugal; mrff@uevora.pt; 4 MED & Departamento de Medicina Veterinária, Escola de Ciências e Tecnologia, Universidade de Évora, Pólo da Mitra, Ap. 94, 7006-554 Évora, Portugal; rmcc@uevora.pt; 5 MED & Departamento de Zootecnia, Escola de Ciências e Tecnologia, Universidade de Évora, Pólo da Mitra, Ap. 94, 7006-554 Évora, Portugal

**Keywords:** Alentejano pig, Bísaro pig, transcriptome, skeletal muscle, meat quality, intramuscular fat, MYH3, MYH7, MAP3K14

## Abstract

**Simple Summary:**

Screening and interpretation of differentially expressed genes and associated biological pathways was conducted among experimental groups with divergent phenotypes providing valuable information about the metabolic events occurring and identification of candidate genes with major regulation roles. This comparative transcriptomic analysis includes the first RNA-seq analysis of the *Longissimus lumborum* muscle tissue from two Portuguese autochthonous pig breeds with different genetic backgrounds, Alentejano and Bísaro. Moreover, a complementary candidate gene approach was employed to analyse, by real time qPCR, the expression profile of relevant genes involved in lipid metabolism, and therefore with potential impacts on meat composition. This study contributes to explaining the biological basis of phenotypical differences occurring between breeds, particularly the ones related to meat quality traits that affect consumer interest.

**Abstract:**

Gene expression is one of the main factors to influence meat quality by modulating fatty acid metabolism, composition, and deposition rates in muscle tissue. This study aimed to explore the transcriptomics of the *Longissimus lumborum* muscle in two local pig breeds with distinct genetic background using next-generation sequencing technology and Real-Time qPCR. RNA-seq yielded 49 differentially expressed genes between breeds, 34 overexpressed in the Alentejano (AL) and 15 in the Bísaro (BI) breed. Specific slow type myosin heavy chain components were associated with AL (MYH7) and BI (MYH3) pigs, while an overexpression of MAP3K14 in AL may be associated with their lower loin proportion, induced insulin resistance, and increased inflammatory response via NF*k*B activation. Overexpression of *RUFY1* in AL pigs may explain the higher intramuscular (IMF) content via higher GLUT4 recruitment and consequently higher glucose uptake that can be stored as fat. Several candidate genes for lipid metabolism, excluded in the RNA-seq analysis due to low counts, such as *ACLY*, *ADIPOQ*, *ELOVL6*, *LEP* and *ME1* were identified by qPCR as main gene factors defining the processes that influence meat composition and quality. These results agree with the fatter profile of the AL pig breed and adiponectin resistance can be postulated as responsible for the overexpression of MAP3K14′s coding product NIK, failing to restore insulin sensitivity.

## 1. Introduction

Alentejano (AL) and Bísaro (BI) are the prevailing autochthonous pig breeds in Portugal. AL is reared in the southern region [1] and shares a genetic closeness with the Iberian pig [2], showing slow growth rates (excluding when under the late “montanheira” fattening regime) and an early and high adipogenic activity [3,4]. BI pigs, on the other hand, common to the northern region, belong to the Celtic line and share ancestors with leaner and highly productive breeds [5]. Furthermore, BI have a lower predisposition for (monounsaturated) fat accumulation when compared to AL pigs, but higher than most commercial lean breeds [6]. These two breeds are well-adapted to the environment, and their production chains provide high quality meat and fermented and dry-cured meat products [3,6].

Fatty acid content and composition are two of the most important factors that influence overall meat quality and consumer preference. A low saturated fatty acid (SFA) content is often desired because increases in this content have been found associated with raising blood cholesterol levels, particularly low-density lipoprotein cholesterol (LDL-c), increasing the risk of heart diseases [7]. On the other hand, increased monounsaturated fatty acid (MUFA) and essential polyunsaturated fatty acid (PUFA) contents are useful in decreasing LDL-c levels while increasing high density lipoprotein-cholesterol, and therefore reducing the risk of heart diseases [8]. Meanwhile, today’s consumers are more aware of the specific nutritional value associated with meat, and that increased fat content contributes to better meat flavour while improving tenderness and juiciness, particularly when it occurs as intramuscular fat (IMF) at levels higher than 2.5% [9,10,11]. These fat stores can appear associated to intramuscular adipocytes or as droplets in the myofiber cytoplasm and can hold excess phospholipids, triacylglycerols, and cholesterol [12,13]. Although IMF content in AL pigs is generally higher than that in BI or other leaner highly productive breeds, their levels tend to fluctuate among several studies, ranging from 3 to 8%, mainly due to feeding and rearing conditions [3,14]. IMF content of the *Longissimus lumborum* muscle (LL) is determined and regulated by multiple metabolic pathways and is associated with the expression of genes involved in carbohydrate and lipid metabolism, cell communication, binding, response to stimulus, cell assembly, and organisation [15,16].

When compared to those of highly productive breeds, AL carcasses present a lower lean meat content (ranging from 37.5 to 51%) due to the above mentioned higher adipogenic activity and lipid deposition [3,14] while BI carcasses yield a moderate lean meat content (from 46 to 51%) [6]. In the *Longissimus lumborum* muscle, AL pigs present a high MUFA level (48–58%), particularly rich in oleic acid, and a low SFA content (35–44%) [3,17]. Studies regarding BI fat composition are scarce but show that MUFA levels (44–47%) [6,18] are lower and SFA levels (33–40%) similar or slightly lower than in AL pigs. Therefore, and when we consider the effects that unsaturated and saturated fatty acids have on consumers’ health, BI pigs seem to display a slightly better balance of the unsaturated to saturated fatty acids ratio than AL pigs. On the other hand, higher PUFA levels are found in BI when compared to AL or improved genotypes, attaining 10% or higher [3,6,19].

RNA-seq experiments take advantage of next-generation sequencing developments to enable a quantitative screening for distinct gene expression patterns in individuals at the transcriptome level. Interpretation of differentially expressed genes (DEGs) and associated biological pathways provide valuable information about the metabolic events occurring and identification of candidate genes with major regulation roles. This comparative transcriptomic analysis includes the first RNA-seq analysis of the *Longissimus lumborum* muscle tissue from these two autochthonous pig breeds. Moreover, a complementary candidate gene approach was employed to analyse, by real time qPCR, the expression profile of relevant genes involved in lipid metabolism. This study can contribute to explain the biological basis of the phenotypical differences occurring between these breeds, particularly the ones related to meat quality traits that affect consumer interest.

## 2. Materials and Methods

The experiment was conducted in accordance with the regulations and ethical guidelines of the Portuguese Animal Nutrition and Welfare Commission (DGAV, Lisbon, Portugal) following the 2010/63/EU Directive. Staff members of the team involved in animal trials were certified for conducting live animal experiments by the Directorate of Animal Protection (DSPA, DGAV, Lisbon, Portugal).

### 2.1. Sampling and FA Profiling

Ten purebred male castrated AL and BI pigs (*n* = 5 for each breed) were reared in a traditional free ranged system and individually fed commercial diets at estimated ad libitum consumption from ~65 kg body weight until slaughter (~150 kg), as previously described [14]. *Longissimus lumborum* muscle samples were obtained at slaughter, snap frozen in liquid nitrogen and maintained at −80 °C until further use.

Total lipids were obtained using a Soxtherm automatic apparatus (S206 MK, Gerhardt). The respective FA profile was determined from the lipid extract, according to a previously described method [20]. After methylation [21], the FA samples were identified by GC-MS QP2010 Plus (Shimadzu Corporation, Kyoto, Japan) and a 60 m × 0.25 mm × 0.2 µm fused silica capillary column (Supelco SP™ 2380, Baltimore, PA, USA). The chromatographic conditions were as follows: injector and detector temperatures were set at 250 and 280 °C, respectively; helium was used as the carrier gas at 1 mL/min constant flow; the initial oven temperature of 140 °C was held for 5 min, increased at 4 °C/min to 240 °C and held for 10 min. MS ion source temperature was set at 200 °C and interface temperature at 220 °C. Identification of FAMEs was based on the retention time of reference compounds.

### 2.2. RNA Extraction and Sequencing

Total RNA was isolated from 50–100 mg samples of LL following Ambion^®^ RiboPure™ Kit (Thermo Fisher Scientific, Waltham, MA, USA) instructions. Total extracted quantity obtained was measured using NanoDropTM 1000 spectrophotometer (Thermo Fisher Scientific, Waltham, MA, USA), while RNA quality was assessed with an Agilent 2100 BioanalyzerTM (Agilent Technologies, Santa Clara, CA, USA) following Agilent RNA 6000 Nano Kit instructions, along with NanoDropTM 1000 260/280 and 260/230 coefficients.

RIN values ranged from 7.8 to 9.0 with an average of 8.42. The total RNA was diluted into a concentration of 100 ng/µL and 3 µg were sent for stranded paired-end mRNA-seq sequencing in *Centro Nacional de Análisis Genómico* (CNAG-CRG, Barcelona, Spain) on a HiSeq2000 sequence analyser (Ilumina, Inc., San Diego, CA, USA). Currently, several RNA-seq experiments are performed at a low replication level and several publications suggest that a minimum of 2–3 replicates can be considered [22,23,24].

### 2.3. Quality Control, Mapping and Assembly

Generated Fastq files were analysed using FastQC (version 0.11.8) for quality confirmation [25] and Trim Galore (version 0.5.0) [26] was used to trim sequence reads for adapters, poli-A and poli-T tails. Low quality nucleotides (Phred Score, Q < 20) as well as short length reads (<40) were also removed, and the remaining reads were mapped to the reference pig genome version Sscrofa11.1 (Ensembl release 94) using HISAT2 version 2.1.0 [27]. Samtools−1.9 [28] was used to convert the obtained SAM files to BAM. Read counting and merging was performed with HTSeq-count version 0.11.1 [29].

### 2.4. Differential Expression Analysis

The obtained Gcount files were analysed using the R package DESeq2 [30], which estimates gene expression levels by counting total exon reads for the statistical analysis. Normalised counts were filtered for a minimum of 50 reads per group, and genes were considered as differently expressed when featuring an FDR lower than 0.05. The raw data shown and interpreted in this publication have been deposited in NCBI’s Gene Expression Ombibus [31] and are available through the GEO Series accession number GSE159817 (https://www.ncbi.nlm.nih.gov/geo/query/acc.cgi?acc=GSE159817, accessed on 1 March 21).

### 2.5. Enrichment Analysis

An enrichment analysis based on the functional annotation of the differentially expressed genes was performed using the Ingenuity Pathways Analysis software (IPA; QIAGEN, Redwood City, CA, USA) to better understand their biological implications within the muscle tissue context. The list of DEGs (q < 0.05, log2FC ≤ −0.7 V log2FC ≥ 0.7) was uploaded into the software and converged with IPA’s own library (Ingenuity Pathway Knowledge Base) to determine biologically pertinent information such as activated pathways, functions and regulators [32].

### 2.6. Real Time qPCR

Real Time quantitative PCR was performed to validate the data obtained by RNA sequencing (*MYH3*, *MYH7*, *TNNT1*, *MAP3K14*, *WDR91*, *FBXO32* and *FASN*) and explore other meat quality candidate genes outside the RNA-seq output (*ACACA*, *ACLY*, *ADIPOQ*, *ELOVL6*, *LEP*, *ME1* and *SCD*). Additional information on the selected primers can be found in the Supplementary Table S1. *MAP3K14*, *MYH3*, *TNNT1* and *WDR91* primers were designed using Primer3 version 0.4.0.

Previously extracted total RNA from the experimental animals was reverse transcribed in 20 µL reactions using Maxima^®^ First Strand cDNA Synthesis Kit for RT-qPCR (Thermo Scientific, Waltham, MA, USA) following manufacturer’s instructions.

The reaction mixes containing 12.5 μL of NZY qPCR Green Master Mix (2×) (NZYtech, Lisbon, Portugal), 0.3 μM of each sense primer and 12.5 ng of cDNA per sample were prepared in 96-well plates and run in a LineGene9600 Plus system (BIOER, Hangzhou, China). Standard PCRs were performed to confirm amplicon sizes, no-cDNA negative controls were performed within every plate and three replicates were performed per target sample. Cycling conditions included an initial 10 min holding denaturation stage at 95 °C, followed by 40 amplification cycles of 15 s denaturation at 95 °C and 50 s at 60 °C. To test PCR specificity a dissociation curve was added as a final step to the program comprising a single cycle at 95 °C (15 s) followed by 60 °C (60 s), and a ramp-up 0.2 °C/s to 95 °C for 15 s with acquired fluorescence. Moreover, PCR efficiency was predicted by standard curve calculation using five points of cDNA dilutions (1:4; 1:8; 1:16; 1:32; 1:64). *ACTB, HSPCB*, *RPL19* and *TOP2B* were the most stable genes tested using the geNorm software [33] and were, therefore, chosen as endogenous control genes for target normalisation (m < 1.5). Cycle Threshold values were regressed on the log of the previously constructed template cDNA curve.

### 2.7. Statistical Analysis

Results are presented as the mean ± SE. All data were tested for normality using the Shapiro–Wilk test. Individual data of growth, plasma, carcass and meat quality traits were analysed by one-way ANOVA with genotype as the main effect. For the carcass data, hot carcass weight was included as a covariate in the model. The SPSS Statistics software (IBM SPSS Statistics for Windows, v24.0; IBM Corp., Armonk, NY, USA) was used for data analysis. Mean differences were considered significant when *p* < 0.05, and values between 0.05 and 0.10 were considered trends.

To determine if the gene expression values were significantly different between the experimental groups, a student’s t-test was executed using IBM SPSS Statistics software (IBM SPSS Statistics for Windows, Version 24.0. Armonk, NY: IBM Corp.) with an established significance level of *p* < 0.05. Equal variances of the samples were checked with Levene’s Test for Equality of Variances with values lower 0.05 not considered as equal variances and another Independent Samples Test was performed assuming no equal variances. Equal variances were not assumed for *WDR91* (F = 7.643; *p* = 0.024) and *LEP* (F = 8.929; *p* = 0.017). Pearson correlation coefficients and associated *p*-values were also estimated.

## 3. Results and Discussion

### 3.1. Productivity of Alentejano and Bísaro Breeds–Summary of Carcass Traits and FA Proportion

In previous studies, we assessed data of AL, BI and their reciprocal crosses, regarding their blood parameters, as well as their productive and meat quality traits, including the *Longissimus lumborum*. Briefly, BI showed significantly better carcass traits than AL and intermediate values were obtained in the crossed pigs (Table 1) [14].

In this study, five randomly selected individuals from pure AL and BI breeds were chosen for transcriptome analysis through RNA-seq. AL pigs averaged a total of 150.6 days on trial with an average daily gain (ADG) of 582 g/d while BI averaged 135.2 days on trial (*p* = 0.273) and an ADG of 656 g/d (*p* = 0.297). At 150 kg and when compared to BI pigs, AL showed higher plasma total protein (69.8 vs. 64.4 g/L, *p* < 0.05), urea (6.9 vs. 5.6 mmol/L, *p* < 0.05) and total cholesterol (2.66 vs. 2.23 mmol/L, *p* < 0.05). Average backfat thickness was also significantly higher in AL (7.9 vs. 4.3 cm, *p* < 0.001) when compared to BI pigs. Regarding the LL muscle, AL pigs presented a lower loin proportion (3.63 vs. 5.14%, *p* < 0.05) but higher total IMF (7.3 vs. 5.7 g/100 g, *p* < 0.01) when compared to BI pigs. Several blood parameters from these pigs are listed in Tables S2 and S3.

The FA analysis identified oleic acid (C18:1 n-9) as the most represented fatty acid in the LL muscle of both breeds, with AL showing higher values when compared to BI (35.00 vs. 29.91%, respectively, *p* < 0.05). The total MUFA (C16:1 n-7, C18:1 n-7, C18:1 n-9, and C20:1 n-11) proportion was also higher in AL pigs (45.42 vs. 39.47%, *p* < 0.05). Regarding SFAs, palmitic acid (C16:0) was the most represented, with similar proportion in both breeds (22.28 vs. 21.22%, *p* = 0.139) while stearic acid (C18:0) was lower in AL pigs (10.77 vs. 12.38%, *p* < 0.01) when compared to BI. Total SFA (C10, C14, C16, C17, C18, and C20) proportion was also comparable between breeds (34.68 vs. 35.24%, *p* = 0.587). Finally, in respect to polyunsaturated fatty acids (PUFA), linoleic acid (C18:2 n-6) was the one most represented in both breeds, but a lower proportion was found in AL (12.06 vs. 15.17%, *p* < 0.01). Total PUFA (C18:2 n-6, C18:3 n-3, C18:3 n-6, C20:2 n-6, C20:3 n-6, C20:4 n-6, C20:5 n-3, C22:4 n-6, C22:5 n-3, and C22:5 n-6) proportion was also lower in AL when compared to BI pigs (19.90 vs. 25.29%, *p* < 0.01).

### 3.2. Mapping and Annotation

More than 750 million reads were initially obtained with an average number of sequenced reads over 39 million per sample. Read length was 76 base pairs. All samples shared an average associated quality score proximate to 40 and total GC content ranged from 53 to 56%. HISAT2 mapping of treated sequences was successfully achieved with an overall 97% of alignment rate, similar to a previous study conducted by our team with adipose tissue [34] but slightly higher than several previous pig transcriptome studies [35,36,37,38,39,40].

### 3.3. Differential Gene Expression with DESeq2 and qPCR Comparison

More than 25 k genes were firstly detected in the muscle tissue with around 3.6 k coding genes obtained after the initial filtering. The overall top five most expressed genes included rabaptin (RAB GTPase Binding Effector Protein 2, *RABEP2*), septin 1 (*SEPTIN1*), a non-identified coding gene (ENSSSCG00000036235), cathepsin F (*CTSF*) and SplA/ryanodine receptor domain and SOCS box containing 2 (*SPSB2*). These genes averaged a total read count per LL sample between 340 k (*RABEP2*) and 89 k (*SPSB2*).

A total of 49 genes were found to be differentially expressed (DE), with 34 being overexpressed in AL (log2 FC ≥ 0.7, q < 0.05) and 15 in BI (log2 FC ≤ −0.7, q < 0.05). In our LL samples, the most overexpressed gene in AL was the Cyclin and CBS domain divalent metal cation transport mediator 3 (*CNNM3*) (log2 FC = 4.487, q < 0.01), while in BI the most overexpressed gene was Stathmin 3 (*STMN3*) (log2 FC = −4.033, q < 0.01). The full detailed list of DEG’s can be found in Table S4.

**Figure 1:**
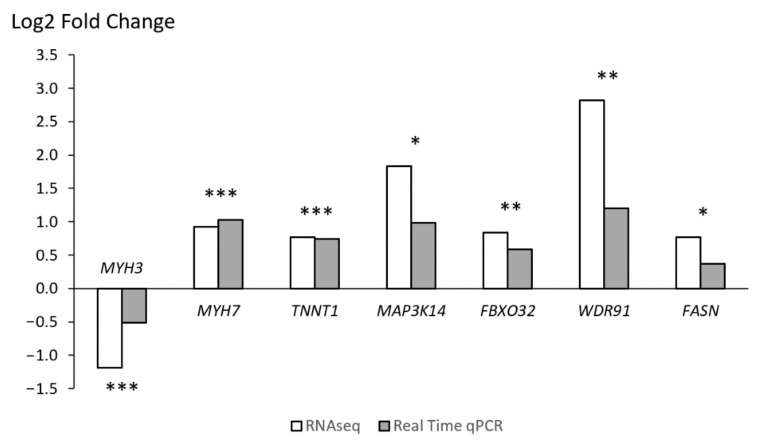
Gene expression comparison of *MYH3* (Myosin heavy chain 3), *MYH7* (Myosin heavy chain 7), *TNNT1* (Troponin T1), *MAP3K14* (Mitogen-activated protein kinase kinase kinase 14), *FBXO32* (F-Box Protein 32), *WDR91* (WD Repeat Domain 91) and *FASN* (fatty acid synthase) with RNA-seq and Real Time qPCR of the *Longissimus lumborum* of Alentejano (AL) and Bísaro (BI) pigs (*n* = 10). Positive values indicate overexpression in AL and negative values overexpression in BI. Pearson correlation values fluctuated between 0.93 (*MYH3*) and 0.58 (*FASN*). Significance of the correlation: *** *p* < 0.001, ** *p* < 0.01, * *p* < 0.05.

Myosin heavy chain 3 (*MYH3*) was found significantly overexpressed in BI in the RNAseq study (log2 FC = −1.191, q < 0.05) but only a numerical difference was observed when analysed through qPCR (log2 FC = −0.510, *p* = 0.150) (Figure 1). This gene is responsible for encoding part of a contractile protein, myosin, which is fundamental for the appropriate function of myofilaments, particularly the sarcomeres of striated/skeletal muscles. *MYH3* is also recognised as an embryonic myosin heavy chain, due to being mainly overexpressed during early mammalian development [41]. In mice, deletion of *MYH3* can induce postnatal changes in muscle fibre number, size, and type, while also deregulating other genes involved in muscle differentiation [41]. Within a species, muscle fibre composition is affected by genetics and several environmental factors while the different fibre types are associated with the four different myosin heavy chain isoforms, or their mixture [42,43]. In 2015, Hou [44] found significantly higher levels of MYH3 in leaner Landrace pigs when compared to fatter indigenous Chinese pig breeds. This author also suggested an association of the overexpression of *MYH3* with the larger diameter in muscle fibres of Landrace pigs. More recently, this gene was identified as a quantitative trait locus with considerable effect on IMF deposition, myofiber type differentiation and reddish meat colour (a* from the CIELAB system) on skeletal muscle of Korean native pigs due to a variant in its promotor region [45]. The impact on a* (red coloration) is mainly associated with the higher content of slow/type 1/oxidative myofibers which were, in turn, associated to an overexpression of the *MYH3* gene. BI’s a* values in LL samples are generally lower when compared to AL pigs (7.8 vs. 10.3, *p* < 0.01) [14] but slightly higher when compared to leaner breeds such as Landrace (5.63), Duroc (7.32) and Yorkshire (6.91) [46]. Similarly, and as mentioned before, BI pigs tend to accumulate less IMF than AL pigs (5.7 vs. 7.3 g/100 g, *p* < 0.01) but significantly more than extensively selected lean breeds. Therefore, our results indicate that in AL pigs other transcription regulatory mechanisms than MYH3 signalling play a bigger and more powerful role in influencing these complex traits since frequently meat quality traits depend on multiple mechanisms determined by numerous loci. Nevertheless, MYH3 signalling may explain the moderate to high levels of redness and IMF presented by BI pigs when compared to leaner breeds. These traits contribute to an improved meat quality and are due to possibly higher content of oxidative (red) muscle fibres, although muscle fibre types were not measured in this study.

Surprisingly, a gene encoding for another myosin heavy chain component, *MYH7*, showed a trend towards AL (log2 FC = 0.921, q = 0.076), a difference boosted by qPCR (log2 FC = 1.025, *p* < 0.01). *MYH7* is also a molecular marker for slow/type 1/oxidative muscle fibres but is generally associated with the heavy chain subunit of cardiac myosin, although it can also be found expressed in skeletal muscle tissue [47].

Another important factor regulating muscle contraction is troponin T1 (*TNNT1*), a subunit of troponin which, as *MYH7*, tended to be overexpressed in AL either in RNA-seq (log2 FC = 0.764, q = 0.091) as in qPCR (log2 FC = 0.738, *p* = 0.070). *TNNT1* expression is also specific of slow skeletal muscle fibres in vertebrates and regulates muscle contraction through the troponin complex which is mediated by calcium concentration in the cells [48].

In our results, we associate both MYH7 as well as TNNT1 signalling in AL with an increase in the differentiation of slow muscle fibres, and to a stronger extent to what occurs in BI pigs through MYH3 signalling. No markers for fast skeletal muscle fibres were detected in the *p* < 0.05 or either *p* < 0.1 significance range suggesting identical presence of this fibre type in both breeds. On the other hand, red fibre type presence is suggested to be linked to MYH3 signalling in BI pigs, and MYH7 and TNNT1 signalling in AL pigs. Consequently, a higher presence of slow muscle fibres in AL pigs is associated with inducing reddish meat with increased IMF content and overall meat quality.

The SET and MYND Domain-Containing Protein 5 (SMYD5) is a member of the SMYD family of methyltransferases which play a crucial role in manipulating gene expression through post translational modifications on unique histone residues and other proteins [49]. SMYD proteins have been recognised as key regulators in skeletal and cardiac muscle development and function, though little is known about the specific roles linked to *SMYD5* [50]. Some studies also suggest a role of *SMYD5* in stimulating an anti-inflammatory response in *Drosophila* [51] and regulation of hematopoiesis in zebrafish [52]. In our data, *SMYD5* was found significantly overexpressed in BI pigs (log2 FC = −2.171, q < 0.01) which agree with the higher ability of this breed to increase muscle tissue when compared to AL pigs, as well as with our previously proposed obesity-induced chronic inflammation state, particularly in AL pigs [34].

Another determining factor of histone modification and transcriptional regulation found in our results was the gene encoding for lysine demethylase 2B (*KDM2B*), found overexpressed in AL pigs (log2 FC = 1.074, q < 0.01). KDM2B is generally associated with the demethylation of histones H3K4, H3K36, and H3K79, and repression of transcription. Recently, KDM2B was suggested to demethylate the non-histone target serum response factor (SRF), consequently inhibiting skeletal muscle cell differentiation and myogenesis [53]. On the other hand, KDM2B has also been heavily linked with an increased inflammatory response through epigenetic regulation of interleukin 6 [54]. Overexpression of *KDM2B* in AL pigs supports our previous hypotheses of lower muscle deposition and higher inflammatory state occurring in AL pigs when compared to BI pigs.

The lymphocyte specific protein 1 (LSP1) was found to be significantly overexpressed in BI pigs (log2 FC = −1.052, q < 0.01). *LSP1* encodes for an intracellular F-actin binding protein that participates in functions such as cell migration and signalling, particularly by regulating the recruitment of circulating leukocytes to inflamed sites [55]. In 2013, Ehrlich [56] first suggested the role of *LSP1* in influencing skeletal muscle development. Results from his work consistently associated an exceptional myogenic differential methylation in various subregions of the *LSP1* gene through binding to the myogenic transcription factor MYOD, particularly in the murine region of the gene. Consequently, DNA methylation status of LSP1 may prove key for alternative promoter usage and stimulating highly specific myogenic factors in BI pigs.

WD Repeat Domain 91 (*WDR91*) was found to be significantly overexpressed in AL pigs through RNAseq (log2 FC = 2.818, q < 0.01) as well as qPCR (log2 FC = 1.197, *p* < 0.05). This gene encodes for a protein that is known to negatively regulate a core subunit of the phosphoinositide 3-kinase (PI3K) complex. This complex is involved in the regulation of several functions including cell growth, proliferation, and differentiation. PI3K signals a network that ultimately leads to mTOR activation [57,58]. Continuous inhibition of this pathway in AL pigs suggests a decrease in overall protein synthesis which agrees with the lower muscle deposition when compared with BI pigs. Furthermore, PI3K activity can act as a molecular switch to regulate correct insulin signalling and activation [59]. Downregulation of this pathway through WDR91 agrees with the proposed lower insulin sensitivity in AL pigs via PI3K inhibition. *WDR91* has also been previously associated as a potential epigenetic regulator of skeletal muscle stem cells in adult mice, which are crucial for the maintenance and regeneration of adult skeletal muscles [60].

The role of epigenetics in the modulation of myogenesis is a current topic of increasing scientific interest, particularly due to the development of new methods that profile methylation. Mammalian DNA methylation is known to regulate the expression of specific target genes through silencing or upregulation, controlling the direction of major metabolic pathways [56]. Our results suggest a solid presence and influence of these mechanisms, particularly through activation of *SMYD5* and *LSP1* in BI and *KDM2B* and possibly *WDR91* in AL pigs, in stimulating BI muscle growth and inhibiting skeletal muscle differentiation in AL pigs, respectively.

The gene that encodes for the F-box protein 32 (*FBXO32*), also known as muscle atrophy F-box protein (*MAFbx*), tended to be overexpressed in AL pigs (log2 FC = 0.833, q = 0.094) which was confirmed by qPCR (log2 FC = 0.587, *p* = 0.098). Protein degradation in skeletal muscles is primarily mediated by the ubiquitin proteasome pathway, particularly muscle specific ubiquitin ligases, of which FBXO32 is proposed to have a central role in inducing proteolysis [61,62]. Increased skeletal muscle deposition demand higher protein levels which are dependent on the balance between protein synthesis and its degradation rates. Both, protein synthesis and proteolysis, are irreversible processes so that their resulting products, either proteins or aminoacids, do not influence the rates at which both processes take place [63,64]. Our results suggest a higher impact of proteolysis over protein synthesis in shifting this balance. Furthermore, overexpression of *FBXO32* is suggested to negatively modulate protein abundance on the skeletal muscle tissue in AL pigs and, consequently, limit new muscle growth when compared to BI pigs, justifying the lower loin weight in AL pigs.

The mitogen-activated protein kinase kinase kinase 14 (*MAP3K14*) was to be found significantly overexpressed in AL with RNA-seq (log2 FC = 1.829, q < 0.01), a result also confirmed with qPCR (log2 FC = 0.983, *p* < 0.05). *MAP3K14* is a gene encoding for a serine/threonine protein-kinase (NF-*κ*B-inducing kinase, NIK) that binds and transcriptionally regulates the expression of a number of molecules such as proinflammatory cytokines and chemokines [65]. High levels of MAP3K14 have previously been associated with skeletal muscle catabolism and atrophy [66], through increased expression of myostatin and decreased MyoD, which can be associated with the reduced loin proportion in the carcass of AL pigs when compared to that observed in BI (3.63 vs. 5.14%, *p* < 0.05). Several studies have also previously proposed the linkage of NIK overexpression with induced skeletal muscle insulin resistance and chronic inflammation development [67,68], in agreement with our previous suggestion of lower insulin sensitivity of the AL breed [34]. MAP3K14 has also been previously reported as a candidate gene to control feed efficiency in Duroc pigs [69] and its overexpression in AL agrees with the higher feed conversion ratios estimated in our AL and BI pigs (5.45 vs. 4.30 kg/kg, *p* < 0.05). Furthermore, in the hepatic tissue of obese mice, MAP3K14 has been also shown to reduce lipid oxidation via inhibition of peroxisome proliferator-activated receptor alpha (PPARA) [70]. While effects on the muscle tissue remain unclear in the current literature, a similar outcome occurring in the skeletal muscle of our pigs would agree with the fatter profile of AL’s meat.

The ATPase H+ transporting V1 subunit C1 (*ATP6V1C1*) encodes for a component of vacuolar-type proton-translocating ATPase (V-ATPase) which is responsible for mediating the acidification of numerous intracellular components and was found to be overexpressed in AL pigs (log2 FC = 0.760, q = 0.046). This subunit C1 of V-ATPase is highly expressed in osteoclasts which participate in the breakdown of bone tissue [71] and may contribute to the higher bone mass found in BI when compared to AL pigs [72]. On the other hand, V-ATPase activity upregulated by ATP6V1C1 can activate the mTOR pathway which is involved in the regulation of multiple processes that lead to protein synthesis and muscle development [73].

Fibroblast growth factor (FGF) signalling can produce numerous beneficial effects on metabolic associated morbidities. FGFs are key for skeletal muscle regeneration and higher abundance is also associated with a higher presence of connective tissue [74,75]. FGFs signal via FGF receptors, requiring the binding of specific klotho proteins. Klotho Beta (*KLB*) is a cell-surface protein coding gene that was found overexpressed in AL pigs (log2 FC = 1.232, q < 0.01). The KLB product is suggested to be mandatory in the activation of several endocrine FGFs including FGF21, FGF19 and FGF15 [76]. FGF15 and 19 in particular are known to downregulate lipogenesis, bile acid metabolism and feeding response, while promoting cell proliferation [77]. On the other hand, FGF21 promotes insulin sensitivity, energy usage, and consequently weight loss [78]. In view of this, overexpression of KLB in AL pigs is, therefore, an intriguing result since AL pigs are proposed to have lower insulin sensitivity and higher lipid deposition than BI pigs. Nevertheless, other regulatory mechanisms might be influencing this pathway, particularly FGF receptor expression, which may play an important role in limiting FGF21, FGF19 and FGF15 signaling in the muscle tissue of AL pigs.

Surprisingly, the gene encoding for leiomodin 1 (*LMOD1*) which is associated with smooth muscle differentiation and contraction, has been found overexpressed in BI pigs (log2 FC = −0.714, q < 0.05) when compared to AL pigs. In vertebrates, LMOD2 and LMOD3 isoforms are preferably more expressed in skeletal muscle cells than LMOD1 [79]. Higher expression levels of the latter may be linked to hyperthyroidism while low levels have been linked to atherosclerosis in humans [80].

In our data, Casein kinase 1 delta (*CSNK1D*) was found overexpressed in BI pigs (log2 FC = −0.674, q < 0.05) when compared to AL pigs. *CSNK1D* participates in the regulation of several processes through phosphorylation of many distinct protein substrates involved in cell proliferation and differentiation [81]. Additionally, it has also been demonstrated that CSNK1D is decisive in maintaining the accuracy of circadian rhythms in mammals [82]. The circadian clock is known to influence several canonical pathways including mTORC1 activity [83].

Regarding lipid metabolism related genes, prostaglandin E synthase 2 (*PTGES2*) was found to be significantly overexpressed in the fatter AL pigs (log2 FC = 1.551, q < 0.05). Prostaglandin E2 participates in several biological activities, including smooth muscle function, body temperature regulation, pain induction and stimulation of bone resorption [84]. PUFAs can affect prostaglandins by serving as substrates and competitive inhibitors for their synthesis [85]. PTGES2 mediates the synthesis of prostaglandin E2 from arachidonic acid (C20:4 n-6) which may explain the numerically higher proportion of this PUFA in BI pigs.

Prolyl 4-hydroxylase subunit beta (*P4HB*) was found overexpressed in AL pigs (log2 FC = 1.171, q < 0.05). *P4HB* encodes for a highly abundant and multifunctional protein involved in the catalysis of the formation, breakage, and rearrangement of disulphide bonds. Additionally, expression of P4HB has been linked with the biology of cytosolic lipid droplets in specific human enterocytes [86].

The gene encoding for 5’-aminolevulinate synthase 1 (*ALAS1*) was found significantly overexpressed in AL (log2 FC = 2.322, q < 0.05). The mitochondrial enzyme produced primarily catalyses the rate-limiting step in heme biosynthesis and its deficiency has previously been associated with acute hepatic porphyrias [87]. *ALAS1* is ubiquitously expressed, commonly regarded as a housekeeping gene, since every nucleated cell must synthesise a heme group for respiratory cytochromes [87]. More recently, ALAS1 has been associated with lipid metabolism regulation through peroxisome proliferator-activated receptor alpha (PPARA). A study by Rakhshandehroo [88] has demonstrated higher expression levels of *ALAS1* specifically induced by PPARA in human hepatocytes.

### 3.4. Functional Analysis

A total of 475 biological functions (*p* < 0.05) were retrieved by the IPA software for our dataset (Table 2). Four functions achieved a z-score enabling prediction of the activation direction, namely quantity of cells (z-score = 2.185) and quantity of leucocytes (z-score = 2.152), which were both predicted to be increased in AL, while neuronal cell death (z-score = −2.164) and apoptosis of tumour cell lines (z-score = −2.043) were predicted to be increased in BI.

As expected, *MAP3k14′*s coding product NIK is signalling the noncanonical NF-*k*B pathway, an alternative signalling cascade involved in the recruitment of leukocytes, macrophages, and lymphocytes. Though the respective retrieved z-score was below the threshold, lipid synthesis is suggested to be enhanced in AL which agrees with the previous phenotypical data.

A total of 64 upstream regulators were found related to the dataset (*p* < 0.05, Table S5) though none surpassed the required activation z-score threshold.

**Figure 2:**
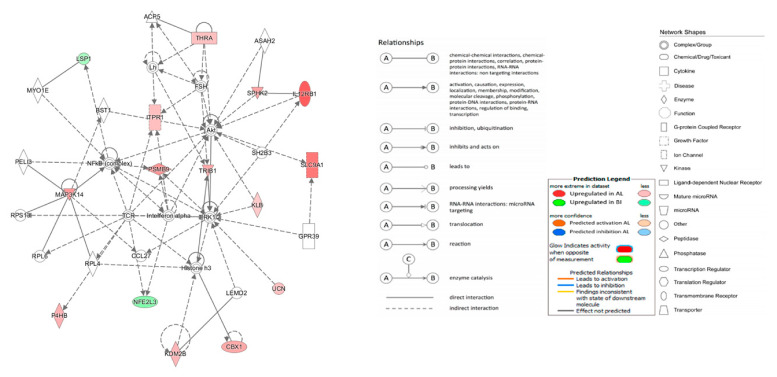
Cellular movement, lipid metabolism and small molecule biochemistry Ingenuity Pathway Analysis (IPA) Network.

Ten networks associated to our gene dataset were identified with IPA. The top network found is represented in Figure 2, resuming 15 focus molecules and a total score of 3. This network illustrates the major involvement of the NF*κ*B complex (mediated by MAP3K14), Histone h3 complex (mediated by KDM2B), extracellular-signal-regulated protein kinase 1/2 (ERK 1/2, mediated by KLB) as main contributors for numerous gene interactions related to cell proliferation, differentiation and biochemistry.

### 3.5. Candidate Gene Expression Analysis with Real Time Quantitative PCR

**Figure 3:**
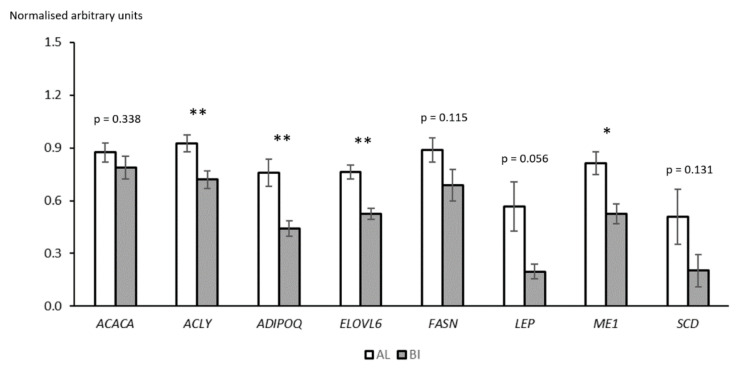
Estimated relative expression of candidate genes involved in lipid metabolism in the *Longissimus lumborum* of Alentejano (AL) and Bísaro (BI) pigs (*n* = 5 for each genotype) through Real Time qPCR. *ACACA* (Acetyl-CoA carboxylase alpha), *ACLY* (ATP citrate lyase), *ADIPOQ* (Adiponectin), *ELOVL6* (Elongation of long-chain fatty acids family member 6), *FASN* (Fatty acid synthase), *LEP* (Leptin)*, ME1* (Malic enzyme 1)*, SCD* (Stearoyl-CoA desaturase). Represented values are means of relative gene expression with their respective standard errors represented by vertical bars. Significance: ** *p* < 0.01, * *p* < 0.05.

A panel including the most relevant known genes involved in lipid metabolism was selected for quantification by RT-qPCR, as those were excluded in the RNAseq analysis due to their low reads counts. Results on these tested genes suggest a much more important role of lipid metabolism in defining the biochemical properties of each breed’s muscle tissue (Figure 3) when compared to our RNA-seq results. Overall, lipogenic related genes were found significantly overexpressed in AL when compared to BI, such as in the cases of *ACLY* (*p* < 0.01), *ELOVL6* (*p* < 0.01), and *ME1* (*p* = 0.01), which agree with the previously mentioned higher IMF content of AL pigs’ muscle tissue. A key gene in the de novo fatty acid synthesis, *FASN*, was only found with a numerical difference towards AL (*p* = 0.115), and the main fatty acid desaturation inducing gene, *SCD*, followed the same trend (*p* = 0.131). Similarly, expression levels of the complex multifunctional enzyme system coded by *ACACA* and responsible for catalysing the limiting step in fatty acid synthesis, was not statistically different between breeds (*p* = 0.338).

Adiponectin, coded by *ADIPOQ*, is widely known to modulate the lipid and glucose metabolisms by activating fatty acid oxidation pathways and increasing blood glucose utilisation, which culminate in the activation of the AMPK pathway and an increase in energy supply [89]. A study by Choudhary [67] demonstrated that NIK overexpression can induce skeletal muscle insulin resistance, while ADIPOQ is responsible for suppressing NIK expression and restoring insulin sensitivity. In our dataset, the gene coding for NIK expression was found overexpressed in AL when compared to BI samples, suggesting an overcompensation in adiponectin levels. This was confirmed by the qPCR results (Log2 FC = 0.783, *p* < 0.01). These results suggest that adiponectin resistance is occurring, particularly in AL pigs, since higher ADIPOQ levels are associated with leaner individuals while lower levels of this cytokine are detected in obese individuals [90]. According to our RNAseq data, ADIPOQ receptors ADIPOR1 and ADIPOR2 were numerically higher in BI, without statistical significance. This may suggest that a potential lower signalling could be occurring in AL. Future investigation on the expression of these receptors, with a higher biological replication, as well as the circulating levels of adiponectin are needed to confirm this hypothesis.

Leptin, coded by *LEP*, is a cell-signalling hormone responsible for appetite regulation by informing the central nervous system when the total fat stored in the body rises. Individuals with high body fat composition exhibit higher levels of leptin, which signals the brain to decrease feeding and increase the use of stocked energy. Leptin is primarily secreted by the main energy store tissue of the body, white adipocytes, but it can also be found expressed in other tissues including skeletal muscle [91,92]. In our trial, LEP presented a tendency to be upregulated in AL pigs (*p* = 0.056) which agrees with the typically fatter composition of this breed when compared to BI. A suggested state of leptin resistance may be occurring in the AL, comparable to what happens with the genetically similar Iberian pig [93].

Our results from AL and BI breeds, particularly the differences in expression levels of lipogenic genes such as *ACLY* and *ME1* suggest that these may play an important role defining the synthesis of new fatty acids, overruling the importance of more central-rolled genes such as *ACACA* and *FASN*. ACLY and ME1 are responsible for catalysing reactions that produce specific non-lipid precursors, including cytosolic acetyl-CoA and NADPH, that can later be used by ACACA and FASN to assemble palmitic acid (C16:0) [94,95]. This fatty acid is the main precursor for the synthesis of stearic acid (C18:0) through ELOVL6, and oleic acid (C18:1 n-9) through SCD. ELOVL6 is responsible for catalysing the reaction that introduces two carbon groups to several SFAs and MUFAs [96] while SCD is accountable for introducing a cis double bond at the delta-9 position in fatty acyl-CoA substrates, including stearic and palmitic acids [97]. Higher expression levels of ELOVL6 and SCD in the loin muscle agree with the previously mentioned higher oleic acid content of AL and may also justify the lower proportion of stearic acid (C18:0) when compared to BI. Nevertheless, we cannot exclude the possibility of more gene regulators being involved in influencing these traits, particularly ones related to fatty acid desaturation since SCD expression was only numerically higher in AL pigs. The generic overexpression of lipogenic related genes in the LL muscle of the AL breed agrees with the higher IMF content when compared to BI pigs. The contribution of lipolytic genes in the regulation of this metabolic balance remains unclear while the higher leptin and adiponectin signalling in the obese AL suggest that these hormones fail to stimulate lipolytic pathways, possibly through post-transcriptional regulation.

### 3.6. Linking Adipose and Skeletal Muscle Tissue Transcriptomes

In a previous study, we explored the genome function of these two local breeds at the adipose tissue level [34]. Several DE genes involved in lipid metabolism were previously detected in adipose tissue through RNAseq but were not detected in muscle tissue, including *ACLY*, *ELOVL6, FASN*, *LEP*, *ME1* and *SCD*. This agrees with the suggestion that lipid metabolism in muscle and adipose tissues are differently regulated [98,99].

Furthermore, the total DEG output (*p* < 0.05) was also much larger in the adipose tissue when compared to the muscle one (458 vs. 49, respectively), with a greater proportion found overexpressed in the AL breed (57 vs. 69%, respectively). This pattern agrees with the lower relevance of muscle tissue in influencing lipid composition through transcriptional and signalling regulation. Perception of adipose tissue as a mere energy storage element is outdated and its functions have been extended to a pivotal endocrine organ that secretes numerous substances that influence homeostasis and metabolism. On the other hand, the combining interactions between skeletal muscle cells and adipocytes have the most impact in defining fat and lean tissue depositions and their respective efficiency rates [100].

Both studies share a total of four DEGs in common, namely chromobox 1 (*CBX1*), integrator complex subunit 11 (*INTS11*), *STMN3* and RUN and FYVE domain containing 1 (*RUFY1*). *CBX1* encodes for a highly conserved protein that binds to methylated histone 3 tails at the lysine 9 residue, acting as an epigenetic agent that modulates chromatin structure and gene expression [101]. This gene was found to be consistently overexpressed in AL pigs in either adipose or LL (log2 FC = 0.954, q < 0.05 vs. log2 FC = 1.300, q < 0.05, respectively) which indicates the relevance of epigenetic mechanisms in regulating gene expression through histone modifications across different tissues, particularly in AL pigs. *INTS11* encodes for the integrator complex subunit 11, an element of the 12-subunit complex that participates in the transcription and processing of small nuclear RNAs [102] and was found consistently overexpressed in BI in both tissues (log2 FC = −1.297, q < 0.01 vs. log2 FC = −1.675, q < 0.01, respectively). On the other hand, *STMN3* encodes for a highly conserved phosphoprotein in vertebrates, generally associated with the deregulation of microtubule dynamics and tubulin sequestering [103]. *STMN3* is commonly associated with various functions in the nervous system and, in our results, presented similar overexpression patterns in BI pigs in both adipose tissue and LL (log2 FC = −2.058, q < 0.01 vs. log2 FC = −4.033, q < 0.01). *RUFY1* is a gene responsible for encoding a protein that binds to several signalling molecules and is suggested to participate in cell polarity and membrane trafficking mediated by small GTPases [104]. Curiously, *RUFY1* was found overexpressed in BI’s adipose tissue and in AL’s *longissimus lumborum* tissue (log2 FC = −1.809, q < 0.01 vs. log2 FC = 1.060, q < 0.05). This suggests that different requirements are needed across tissues concerning *RUFY1′*s contribution towards cellular homeostasis. Additionally, *RUFY1*, also known as *RABIP4*, can modulate intracellular trafficking of the glucose transporter 4 (GLUT4) which plays a crucial role in increasing the transportation of circulating glucose to the cells [105]. Our results suggest that unexpectedly and despite similar circulating glucose levels in both breeds, either before or after slaughter (see Tables S2 and S3), on the adipose tissue higher circulating levels of glucose are taken to BI’s adipocytes when compared to AL pigs, probably to be stored as fat after glucose homeostasis is achieved. This also suggests that the higher fat stores presented in AL when compared to BI pigs [14] are regulated through other mechanisms. On the other hand, in the LL the opposite is suggested to occur, where higher levels of glucose can be used as fuel to power the muscle cell or long-term stored as glycogen. Higher glucose uptake via *RUFY1* and *GLUT4*, in the AL’s muscle tissue may explain the higher IMF content when compared to BI pigs.

Intramuscular fat participates in important regulatory roles in muscle, particularly through insulin-mediated glucose uptake and lipid peroxidation. Oversupply of fat stores is then strongly associated with decreased insulin sensitivity in skeletal muscle [106]. The proposed obesity-induced chronic inflammatory state in AL pigs, caused by exacerbated lipid accumulation, induces expression of pro-inflammatory cytokines and activation of numerous signalling pathways, including the nuclear factor-kappa B (NF*κ*B) pathway, which ultimately are suggested to inhibit insulin signalling and action [107]. Interestingly, while we associated MAP3K14 with the activation of the NF*κ*B pathway in the LL of AL pigs (log2 FC = 1.829, q < 0.01), in the adipose tissue this role seems to be played by MAP3K15 (log2 FC = 1.036, q < 0.05). However, development of insulin resistance involves several complex biological mechanisms that are not fully understood yet. BI’s lower tendency to store fat when compared to AL pigs does not exclude the possibility of decreased insulin sensitivity to be occurring. BI pigs presented significantly higher total collagen content than AL pigs (13.7 vs. 16.3 mg/g, *p* < 0.05) which has previously been positively associated with insulin resistant skeletal muscle tissue in human patients [108].

Overall expression of the selected candidate genes involved in lipid metabolism was similar between adipose and muscle tissues. In short, lipogenic related genes were consistently more expressed in the AL breed across both studies which agrees with the fatter profile of this breed. Higher absolute fold change values were higher in adipose tissue though more significant differences between breeds were observed in the muscle tissue (Table 3).

Next-generation sequencing techniques such as RNA-seq provide a broad and insightful perspective over a tissue transcriptomics but still struggle with the analysis of low expression data, while differences within high read counts are more easily detected [109]. In our dataset, several key genes involved in lipid synthesis and regulation were found below the cut-off point regarding total read count in the filtering step, which determined their premature withdrawal of the differential expression analysis. FASN, for example, in the adipose tissue scored a great correlation coefficient (0.92, *p* < 0.01) while in the muscle, where it was excluded in the initial filtering (<50 reads per group), presented a much lower, barely significant score (0.58, *p* < 0.05). This is why we suggest that genes with fewer reads detected through RNAseq should be accounted for with care since they arouse more associated noise and differential expression estimations may be biased against low read count values [110,111]. Besides the utility of RNA-seq at a standard read depth (30–60 million reads) for a general overview of the transcriptomic universe, Real Time qPCR provides a more reliable source of information to assess low expressed genes, as our results suggest.

In conclusion, this study intended to determine the nature of the biological events occurring in the muscle of the two main Portuguese autochthonous pig breeds and their interpretation regarding the phenotypical diversity shown by the two breeds. Our data allowed the identification of 49 differentially expressed genes through RNA-seq. Specific myosin heavy chain components have been associated with AL (MYH7) and BI (MYH3) pigs, while an overexpression of *MAP3K14* in AL may be associated with their lower loin proportion, induced insulin resistance, and increased inflammatory response. Our Real time qPCR data acknowledged the importance of several lipogenic genes and regulators, including *ACLY*, *ADIPOQ*, *ELOVL6*, *LEP* and *ME1*, disregarded in the RNA-seq by their lower total read count. These latter results agree with the fatter profile of the AL pig breed and adiponectin resistance may be responsible for the overexpression of MAP3K14′s coding product NIK, failing to restore insulin sensitivity.

## Figures and Tables

**Table 1:** Zootechnical traits and physical–chemical parameters from muscle *Longissimus lumborum* of Alentejano (AL) and Bísaro (BI) pigs slaughtered at ~150 kg LW.

Trait	AL (*n* = 5)		BI (*n* = 5)		Significance
	Mean	SE	Mean	SE	
Days on trial	150.6	5.5	135.2	11.9	0.273
Average daily gain (g/day)	582.4	18.1	656.3	63.8	0.297
Feed conversion ratio (kg/kg)	5.45	0.21	4.3	0.47	0.039
Average backfat thickness (cm) *	7.9	0.4	4.3	0.3	<0.0001
*Longissimus lumborum* (%)	3.63	0.26	5.14	0.52	0.030
Moisture (g/100 g)	70.6	0.2	72.3	0.5	0.008
Total protein (g/100 g)	23.7	0.4	23.4	0.3	0.561
Total intramuscular fat (g/100 g)	7.3	0.2	5.7	0.2	0.001
Myoglobin content (mg/g)	0.83	0.12	0.43	0.04	0.014
Total collagen (mg/g DM)	13.7	0.6	16.3	0.7	0.025
pH (24 h post-mortem)	5.76	0.03	5.50	0.04	0.001
Drip loss (g/100 g)	0.55	0.07	2.25	0.21	<0.0001

Note: * Average of measurements taken between last cervical and first thoracic vertebrae (first rib), and last thoracic and first-lumbar vertebrae (last rib).

**Table 2:** Top functions found with IPA associated with the target molecules in the Deseq2 dataset and their respective predicted activation state in Alentejano pigs (AL).

Functions	*p*-Value	Activation z-Score	Predicted Activation in AL	Target Molecules
Quantity of cells	3.64 × 10^−2^	2.185	Increased	*IL12RB1*, *ITPR1*, *KDM2B*, *LSP1*, *MAP3K14*, *PSMB9*, *SPHK2*, *THRA*, *TRIB1*
Neuronal cell death	1.69 × 10^−2^	−2.164	Decreased	*ITPR1*, *KDM2B*, *P4HB*, *SLC9A1*, *UCN*
Quantity of leukocytes	1.75 × 10^−3^	2.152	Increased	*IL12RB1*, *ITPR1*, *LSP1*, *MAP3K14*, *PSMB9*, *SPHK2*, *THRA*, *TRIB1*
Apoptosis of tumour cell lines	1.32 × 10^−2^	−2.043	Decreased	*ITPR1*, *KDM2B*, *LSP1*, *MAP3K14*, *P4HB*, *SLC9A1*, *SPHK2*, *THRA*
Binding of DNA	1.77 × 10^−2^	1.993	-	*CBX1*, *MAP3K14*, *THRA*, *UCN*
Cell cycle progression	3.52 × 10^−2^	1.964	-	*ANGEL2*, *CSNK1D*, *KDM2B*, *SLFN11*, *SPHK2*, *THRA*
Quantity of macrophages	1.08 × 10^−3^	1.961	-	*IL12RB1*, *LSP1*, *THRA*, *TRIB1*
Quantity of B lymphocytes	9.65 × 10^−3^	1.190	-	*ITPR1*, *MAP3K14*, *SPHK2*, *THRA*
Synthesis of lipid	3.57 × 10^−2^	1.186	-	*MAP3K14*, *PTGES2*, *RUFY1*, *SPHK2*, *TRIB1*

**Table 3:** Candidate gene expression for lipid metabolism comparison between adipose tissue and *longissimus lumborum* in Alentejano (AL) and Bísaro (BI) pigs assessed with qPCR. Positive values indicate overexpression in AL and negative values overexpression in BI.

Genes	Adipose Tissue		*Longissimus lumborum*	
	log2 FC	*p*-Value	log2 FC	*p*-Value
*ACACA*	1.055	0.077	0.150	0.338
*ACLY*	1.601	0.068	0.362	0.017
*ADIPOQ*	−0.685	0.110	0.783	0.007
*ELOVL6*	0.671	0.136	0.540	0.001
*FASN*	1.359	0.100	0.367	0.115
*LEP*	0.929	0.046	1.524	0.056
*ME1*	1.008	0.106	0.627	0.010
*SCD*	1.351	0.087	1.325	0.131

## Data Availability

The results from data analyses performed in this study are included in this article and its tables. Raw sequencing data are available through the GEO Series accession number GSE159817 or from the corresponding author on reasonable request.

## Supplementary Materials

The following are available online at https://www.mdpi.com/article/10.3390/ani11051423/s1, Table S1: Primer design for qPCR, Table S2: Plasma parameters from Alentejano (AL) and Bísaro (BI) pigs at ~150 kg LW, Table S3: Plasma parameters from Alentejano (AL) and Bísaro (BI) pigs slaughtered at 150 kg LW, Table S4: The full detailed list of DEGs, Table S5: Predicted upstream regulators for the set of differentially expressed genes.
