# Supplementary material for: Transcriptomic Profiling of Skeletal Muscle Reveals Candidate Genes Influencing Muscle Growth and Associated Lipid Composition in Portuguese Local Pig Breeds
Source: Animals (Basel). 2021 May 16;11(5):1423. doi: 10.3390/ani11051423 (PMC8156922; doi:10.3390/ani11051423)
Supplement: Supplementary file 1 [file animals-11-01423-s001.zip › Supplementary Table 1_Primer design for qPCR.pdf]

Table S1: Primer design for qPCR.

Genes	Sequence (5'-3')	Product Size (Bp)	Annealing Temp. (ºc)	Accession Number	Reference
ACTB	FW: TCGCACTTCATGATCGAGTTG<br>RV: CGACGGCCAGGTCATCAC	138	60	AY550069	[1]
HSPCB	FW: GGCAGAAGACAAGGAGAAC<br>RV: CAGACTGGGAGGTATGGTAG	131	56	AF288819	[2]
RPL19	FW: GCTTGCCTCCAGTGTCC<br>RV: GTTGGCGTTGGCGATTT	82	62	AF435591	[2]
TOP2B	FW: AACTGGATGATGCTAATGATGCT<br>RV: TGGAAAAACTCCGTATCTGTCTC	137	56	NM_001258386.1	[2]
ACACA	FW: TCCCAGTGCAAGCAGTATG<br>RV: TGCCAATCCACACGAAGAC	211	60	EF618729	[3]
ACLY	FW: GAGGCAGCATCGCAAACTTCAC<br>RV: GGTCTTCCCAACTTCTCCCATC	170	55	NM_001105302.1	[4]
ADIPOQ	FW: CGTTCAGCATTCAGTGTGG<br>RV: TCATTCAATGTTGTGGTAGAGA	178	55	NM214370	[5]
ELOVL6	FW: AGAACACGTAGCGACTCCGAAGAT<br>RV: GACATGCCGACCGCCAAAGATAA	177	60	XM_013978957.1	[6]
FASN	FW: GCAGGCGCGTGATGGGAATGGTG<br>RV: GCCCGAGCCCGAGTGGATGAGCA	206	58	NM_001099930	[6]
FBXO32	FW: TCACAGCTCACATCCCTGAG<br>RV: GACTTGCCGACTCTCTGGAC	167	56	NM_001044588.1	[7]
LEP	FW: GGCCCTATCTGTCCTACGTTGAAG<br>RV: TGGAAGGCAGACTGGTGAGGAT	237	60	NM_213840.1	[6]
MAP3K14	FW: ATGTGACCCATCAAGCTTCC<br>RV: CACCACACAGGGATTCACAG	140	60	XM_003131321.4	-
ME1	FW: GCCGGCTTTATCCTCCTCT<br>RV: TCAAGTTTGGTCTGTATTTTCTGG	223	55	XM_001924333.5	[6]
MYH3	FW: GGGAGCAGAAGAAGAACACG<br>RV: AGGACTTGACCTTCGCTTGA	150	51	XM_013981330.2	-
MYH7	FW: GGTATCGCATCCTGAACCC<br>RV: GCCCTGCCTTGAAGAACAC	144	51	NM_213855.2	[8]
SCD	FW: CCGCCCTGAAATGAAAGATGAC<br>RV: GTAGGCAAACGCCCAGAGCAAG	184	60	NM_213781.1	[4]
TNNT1	FW: GGTCAAGGCAGAACAGAAGC<br>RV: ATCCAATCCGACAGTTCCT	147	58	NM_213748.2	-
WDR91	FW: GTCCGCGAGTACTTGCTCTT<br>RV: CCACAATCTTGTCCACCCGA	106	60	XM_003134652.6	-

1. Guo, X.; Tang, R.; Wang, W.; Liu, D.; Wang, K. Effects of dietary protein/carbohydrate ratio on fat deposition and gene expression of peroxisome proliferator activated receptor γ and heart fatty acid-binding protein of finishing pigs. *Livestock Science* **2011**, *140*, 111-116, doi:http://dx.doi.org/10.1016/j.livsci.2011.02.016.
2. Gu, Y.R.; Li, M.Z.; Zhang, K.; Chen, L.; Jiang, A.A.; Wang, J.Y.; Li, X.W. Evaluation of endogenous control genes for gene expression studies across multiple tissues and in the specific sets of fat- and muscle-type samples of the pig. *Journal of Animal Breeding and Genetics* **2011**, *128*, 319-325, doi:10.1111/j.1439-0388.2011.00920.x.
3. Tan, B.; Yin, Y.; Liu, Z.; Tang, W.; Xu, H.; Kong, X.; Li, X.; Yao, K.; Gu, W.; Smith, S.B., et al. Dietary L-arginine supplementation differentially regulates expression of lipid-metabolic genes in porcine adipose tissue and skeletal muscle. *The Journal of Nutritional Biochemistry* **2011**, *22*, 441-445, doi:10.1016/j.jnutbio.2010.03.012.
4. Gao, Y.; Zhang, Y.H.; Jiang, H.; Xiao, S.Q.; Wang, S.; Ma, Q.; Sun, G.J.; Li, F.J.; Deng, Q.; Dai, L.S., et al. Detection of differentially expressed genes in the longissimus dorsi of Northeastern Indigenous and Large White pigs. *Genet. Mol. Res.* **2010**, *10*, 779-791.
5. Weber, T.E.; Kerr, B.J.; Spurlock, M.E. Regulation of hepatic peroxisome proliferator-activated receptor alpha expression but not adiponectin by dietary protein in finishing pigs. *Journal of Animal Physiology and Animal Nutrition* **2008**, *92*, 569-577.
6. Benítez, R.; Fernández, A.; Isabel, B.; Núñez, Y.; Mercado, E.D.; Gómez-Izquierdo, E.; García-Casco, J.; López-Bote, C.; Óvilo, C. Modulatory Effects of Breed, Feeding Status, and Diet on Adipogenic, Lipogenic, and Lipolytic Gene Expression in Growing Iberian and Duroc Pigs. *International Journal of Molecular Sciences* **2018**, *19*, 22, doi:10.3390/ijms19010022.
7. Liu, Y.; Wang, X.; Leng, W.; Pi, D.; Tu, Z.; Zhu, H.; Shi, H.; Li, S.; Hou, Y.; Hu, C.A. Aspartate inhibits LPS-induced MAFbx and MuRF1 expression in skeletal muscle in weaned pigs by regulating Akt, AMPKα and FOXO1. *Innate immunity* **2017**, *23*, 34-43, doi:10.1177/1753425916673443.
8. Chen, X.; Guo, Y.; Jia, G.; Zhao, H.; Liu, G.; Huang, Z. Arginine Promotes Slow Myosin Heavy Chain Expression via Akirin2 and the AMP-Activated Protein Kinase Signaling Pathway in Porcine Skeletal Muscle Satellite Cells. *J Agric Food Chem* **2018**, *66*, 4734-4740, doi:10.1021/acs.jafc.8b00775.
