# Supplementary material for: Transcriptomic Profiling of Skeletal Muscle Reveals Candidate Genes Influencing Muscle Growth and Associated Lipid Composition in Portuguese Local Pig Breeds
Source: Animals (Basel). 2021 May 16;11(5):1423. doi: 10.3390/ani11051423 (PMC8156922; doi:10.3390/ani11051423)
Supplement: Supplementary file 1 [file animals-11-01423-s001.zip › Supplementary Tables 2 and 3.pdf]

Table S2. Plasma parameters from Alentejano (AL) and Bísaro (BI) pigs at ~150 kg LW

	AL (n=5)		BI (n=5)		Significance
	**Mean**	**SE**	**Mean**	**SE**	
**Total protein (g L^{-1})**	69.8	1.8	64.4	1.4	0.047
**Urea (mmol L^{-1})**	6.9	0.4	5.6	0.2	0.019
**Glucose (mmol L^{-1})**	4.14	0.16	3.99	0.21	0.562
**Triacylglycerols (mmol L^{-1})**	0.48	0.05	0.36	0.03	0.100
**Total cholesterol (mmol L^{-1})**	2.66	0.17	2.23	0.05	0.040
**Cortisol (nmol L-1)**	157.6	20.9	197.1	17.7	0.187

Table S3. Plasma parameters from Alentejano (AL) and Bísaro (BI) pigs slaughtered at 150 kg LW

	AL (n=5)		BI (n=5)		Significance
	**Mean**	**SE**	**Mean**	**SE**	
**Total protein (g L^{-1})**	78.8	1.7	72.0	2.7	0.062
**Urea (mmol L^{-1})**	6.4	0.5	6.5	0.3	0.959
**Glucose (mmol L^{-1})**	9.2	1.1	8.9	0.3	0.819
**Triacylglycerols (mmol L^{-1})**	0.84	0.08	0.70	0.09	0.264
**Total cholesterol (mmol L^{-1})**	2.64	0.18	2.13	0.03	0.025
**Cortisol (nmol L-1)**	410.2	61.4	471.9	58.2	0.486
